# Progressive hyperleukocytosis is a relevant predictive marker for differentiation syndrome, early death, and subsequent relapse in acute promyelocytic leukemia

**DOI:** 10.1038/s41598-019-47937-4

**Published:** 2019-08-15

**Authors:** Jae-Ho Yoon, Hee-Je Kim, Gi June Min, Sung-Soo Park, Young-Woo Jeon, Sung-Eun Lee, Byung-Sik Cho, Ki-Seong Eom, Yoo-Jin Kim, Seok Lee, Chang-Ki Min, Seok-Goo Cho, Jong Wook Lee

**Affiliations:** Department of Hematology, Catholic Hematology Hospital and Leukemia Research Institute, Seoul St. Mary’s Hospital, College of Medicine, The Catholic University of Korea, Seoul, Korea

**Keywords:** Mechanisms of disease, Acute myeloid leukaemia

## Abstract

Acute promyelocytic leukemia (APL) is generally held to have favorable risk, but we have observed a high incidence of early deaths caused by fatal bleeding and differentiation syndrome (DS). We retrospectively analyzed 259 APL patients from 2002 to 2014 who all received all-trans retinoic acid (ATRA) with the support of sufficient transfusions, followed by 4 days of idarubicin. High-risk status was determined as a diagnostic leukocyte count (WBCdx) >10 × 10^9^/L (Sanz criteria). For patients with hyperleukocytosis, we sometimes conducted leukapheresis and also used hydroxyurea and prophylactic dexamethasone. Because we frequently observed patient fatalities from progressive hyperleukocytosis, we also checked the maximum leukocyte count (WBCmax) and stratified patients by their incremental ratios. The 8-week cumulative incidence of early death and DS was 13.5% and 17.8%, respectively. We found that WBCmax correlated better with early death and DS, even in the low-risk group, than WBCdx. Among the patients with WBCdx <10 × 10^9^/L, a WBCmax >43 × 10^9^/L correlated with early death (26.7%) and DS (40.0%). Also, having a WBCdx of 10 to 43 × 10^9^/La that increased to a WBCmax >43 × 10^9^/L correlated with increased early death (33.3%). The multivariate analysis revealed that a WBCmax >43 × 10^9^/L correlated significantly with both early death and DS.

## Introduction

Acute promyelocytic leukemia (APL) is generally held to have favorable risk and be related to good hematological remission, with a long-term overall survival (OS) rate of more than 80% and a lower relapse rate than acute myeloid leukemia^1–6^. The combination of all-trans retinoic acid (ATRA) and anthracycline-based chemotherapy has improved outcomes in APL during the past few decades, and recent trials have shown the safety and efficacy of arsenic trioxide (ATO) and ATRA therapy^7–11^. Nevertheless, we still observe unexpected early deaths from APL, mainly caused by significant bleeding complications, sepsis, or differentiation syndrome (DS), especially in high-risk patients^12–15^.

The National Cancer Institute’s Surveillance, Epidemiology, and End Results study analyzed 1,400 patients and reported an early death rate of 17.3%, which was higher than that in previous reports, and that the rate increased with age, especially in patients older than 55 years^15^. For DS, the Programa Español de Tratamientos en Hematología (PETHEMA) group analyzed 739 patients treated with ATRA and anthracycline-based chemotherapy and reported a DS rate of 24.8%; the rate of the severe form of DS was 12.6%, and it was associated with an increase in mortality^13^. However, a wide range of incidence rates have been reported; many clinical trials have excluded patients with early death, and the criteria used for the diagnosis of DS has varied by study.

Sanz-risk scoring is generally used to identify high-risk APL patients, those who present a high leukocyte count at diagnosis. However, we have frequently observed that patients with progressive hyperleukocytosis from low WBCdx to a high maximum leukocyte count (WBCmax) despite attempts to reduce leukocyte counts by hydroxyurea, cytarabine, or leukapheresis. Diagnostic and peak leukocyte counts and an abnormal creatinine level were reported as predictive factors for DS^13,16^, but other reports found no significant predictive factors^12,17^.

We designed the current study to identify the incidence and clinical manifestations of early death and DS in patients with APL who were mainly treated with ATRA and anthracycline-based chemotherapy. Then we tried to identify factors associated with the progression of leukocyte counts that might affect the development of DS or early death.

## Results

### Baseline characteristics

The main presenting clinical and biological features of all patients (median age: 42 years old [range; 17–72]) at the time of diagnosis are presented in Table 1. The median leukocyte count at diagnosis was 3.73 × 10^9^/L (range; 0.4–172.9), and at peak it was 13.4 × 10^9^/L (range; 0.4–177.0). The median platelet count was 32.0 × 10^9^/L (range; 5.0–216.0), and the median prothrombin time was 69.0% (range; 35.0%–105.0%). Based on Sanz-risk scoring^4,13^, 79 (30.5%) patients were classified into the high-risk group, 108 (41.7%) were in the intermediate-risk group, and 72 (27.8%) were in the low-risk group. All patients showed positive PML-RARA by RT-PCR, but 5 patients showed normal karyotype rather than t(15;17)(q22;q21). Among those with t(15;17)(q22;q21), additional chromosomal aberrations were observed in 77 (29.7%) patients. Of the 190 patients with available *BCR* isotype analyses, *BCR3* was identified in 70 (36.8%) patients, and of the 164 patients with available *FLT3* mutation analyses, 34 had the *FLT3*-ITD mutation and 12 had the *FLT3*-TKD mutation. All but 1 patient received ATRA. Standard chemotherapy using idarubicin and ATRA was administered in 219 (84.6%) patients, whereas ATO and ATRA was administered in 13 (5.0%) patients. Patients presenting a severe comorbidity (n = 26) were treated with ATRA alone because ATO was unavailable at that time. Except for 37 patients who died within 8 weeks without post-induction bone marrow (BM) biopsy, 220 (84.9%) patients achieved complete remission (CR) after induction chemotherapy. The remaining 2 achieved CR after reinduction chemotherapy.

**Table 1:** Baseline characteristics of enrolled patients.

Total n = 259	Number or median value
Age, median (range)	42 (17–72)
Gender, Male	143 (55.2%)
**Laboratory findings at diagnosis**	
Leukocyte count (×10^9^/L) Leukocytes count at peak (×10^9^/L)	3.73 (0.4–172.9) 13.4 (0.4–177.0)
Hemoglobin (g/dL)	8.6 (3.8–15.0)
Platelet (×10^9^/L)	32.0 (5.0–216.0)
Lactate dehydrogenase (LDH, U/L)	688.0 (221.0–5440.0)
Prothrombin time (PT, %)	69.0 (35.0–105.0%)
Partial thromboplastin time (aPTT, sec)	28.0 (20–50)
Fibrinogen (mg/dL)	131.0 (31.0 – 685.0)
Antithrombin III (%)	98.0 (44.0–150.0)
D-dimer (mg/L)	20.0 (1.0–36.0)
**Sanz-risk criteria**	
High	79 (30.5%)
Intermediate	108 (41.7%)
Low	72 (27.8%)
**Karyotype**	
Normal karyotype	5 (1.9%)
t(15;17) alone	177 (68.4%)
t(15;17) with 1 additional karyotype	51 (19.7%)
t(15;17) with ≥2 additional karyotype	26 (10.0%)
***PML-RARA*** **subtype**	
Not assessed	69 (26.6%)
BCR1	120 (46.4%)
BCR3	70 (27.0%)
***FLT3*** **mutation**	
Not assessed	95 (36.7%)
No *FLT3* mutation	118 (45.6%)
*FLT3*-ITD	34 (13.1%)
*FLT3*-TKD	12 (4.6%)
Leukapheresis at initial treatment	26 (18.2%)
>3 times	6 (2.3%)
≤3 times	20 (15.9%)
**Induction chemotherapy**	
ATRA alone	26 (10.0%)
ATRA plus idarubicin	219 (84.6%)
ATRA plus arsenic trioxide	13 (5.0%)
Differentiation syndrome	46 (17.9%)
Median onset day (range)	7.5 (1–46)
**Hematological complete response**	
After 1^st^ induction	220 (84.9%)
After 2^nd^ induction	2 (0.7%)

Abbreviation: BCR, breakpoint cluster region; *FLT3*, Fms-like tyrosine kinase 3; ITD, internal tandem duplication; TKD, tyrosine kinase domain; *ABL*, Abelson murine leukemia viral oncogene; *WT1*, Wilms’ tumor 1; ATRA, all-trans retinoic acid; AML, acute myeloid leukemia.

### Survival outcomes and early events

**Figure 1:**
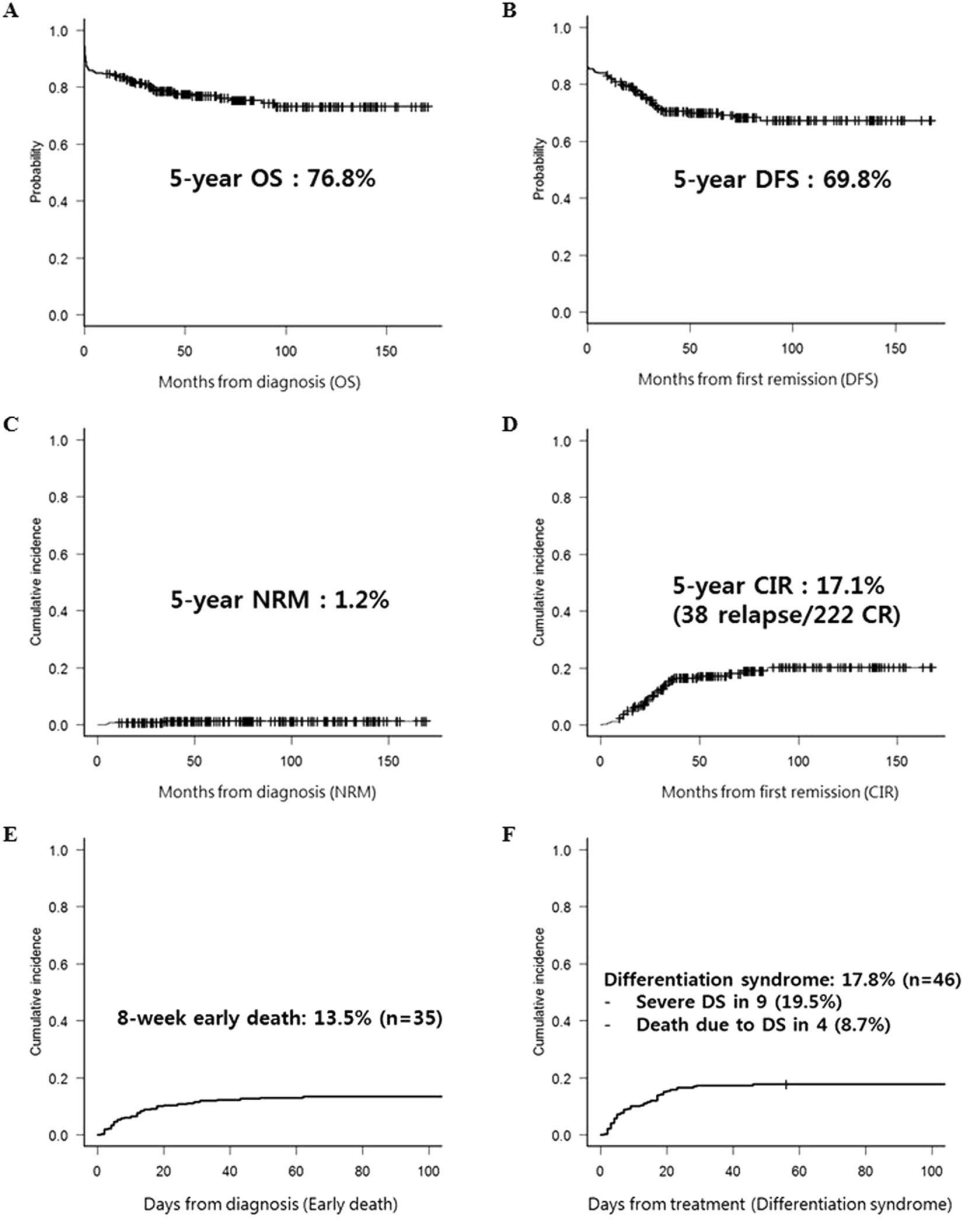
Overall clinical outcomes. (**A**) OS. (**B**). DFS. (**C**) CIR. (**D**). NRM. (**E**) Early death (within 8 weeks). (**F**) DS. Abbreviations; OS, overall survival; DFS, disease free survival; CIR, cumulative incidence of relapse; NRM, non-relapse mortality; DS, differentiation syndrome.

After a median follow-up of 65.4 months (range; 11.1–170.5) for surviving patients, the 5-year OS and disease free survival (DFS) rates of the entire patient cohort were 76.8% (Fig. 1A) and 69.8% (Fig. 1B), respectively. The 5-year non-relapse mortality (NRM) rate of the entire cohort was 1.2% (Fig. 1C). Among the 222 (86.0%) patients achieving CR, the 5-year cumulative incidence of relapse (CIR) rate was 17.1% (Fig. 1D). We defined the time point for early death as 8 weeks, and the 8-week early death rate was 13.5% for all patients (Fig. 1E). The causes of early death were hemorrhage in multiple organs (n = 3), alveolar hemorrhage (n = 13), intracerebral hemorrhage (n = 12), severe DS followed by multiorgan failure (n = 4), and sepsis (n = 3). DS was observed in 46 (17.8%) patients (Fig. 1F), and among them, severe DS was observed in 9 (19.5%), of whom 4 died due to respiratory failure and progressive multiorgan failure.

### Molecular markers and leukocyte counts

Among the 160 patients with both *FLT3* mutation and *BCR* isotype results available, 45 (28.1%) were positive for the *FLT3* mutation, and 54 (33.7%) had the *BCR3* isotype. Of the 45 *FLT3*-positive patients, 26 (57.8%) had the *BCR3* isotype, whereas 87 of the 115 (75.7%) *FLT3*-negative patients had the *BCR1* isotype (*p* < 0.001). Thus, more than half the *FLT3*-positive patients had the *BCR3* isotype, and having either the *BCR3* isotype or *FLT3*-positivity correlated with higher WBCdx (median 9.0 × 10^9^/L, *p* < 0.001) and WBCmax (median 23.1 × 10^9^/L, *p* < 0.001) compared to patients with both the *BCR1* isotype and *FLT3*-negativity (median WBCdx 2.1 × 10^9^/L and WBCmax 6.1 × 10^9^/L). Patients with both the *BCR3* isotype and *FLT3*-positivity (n = 26) had significantly higher WBCdx (median 23.8 × 10^9^/L) and WBCmax (median 45.4 × 10^9^/L) than the other patients.

### Patients with progressive hyperleukocytosis

**Figure 2:**
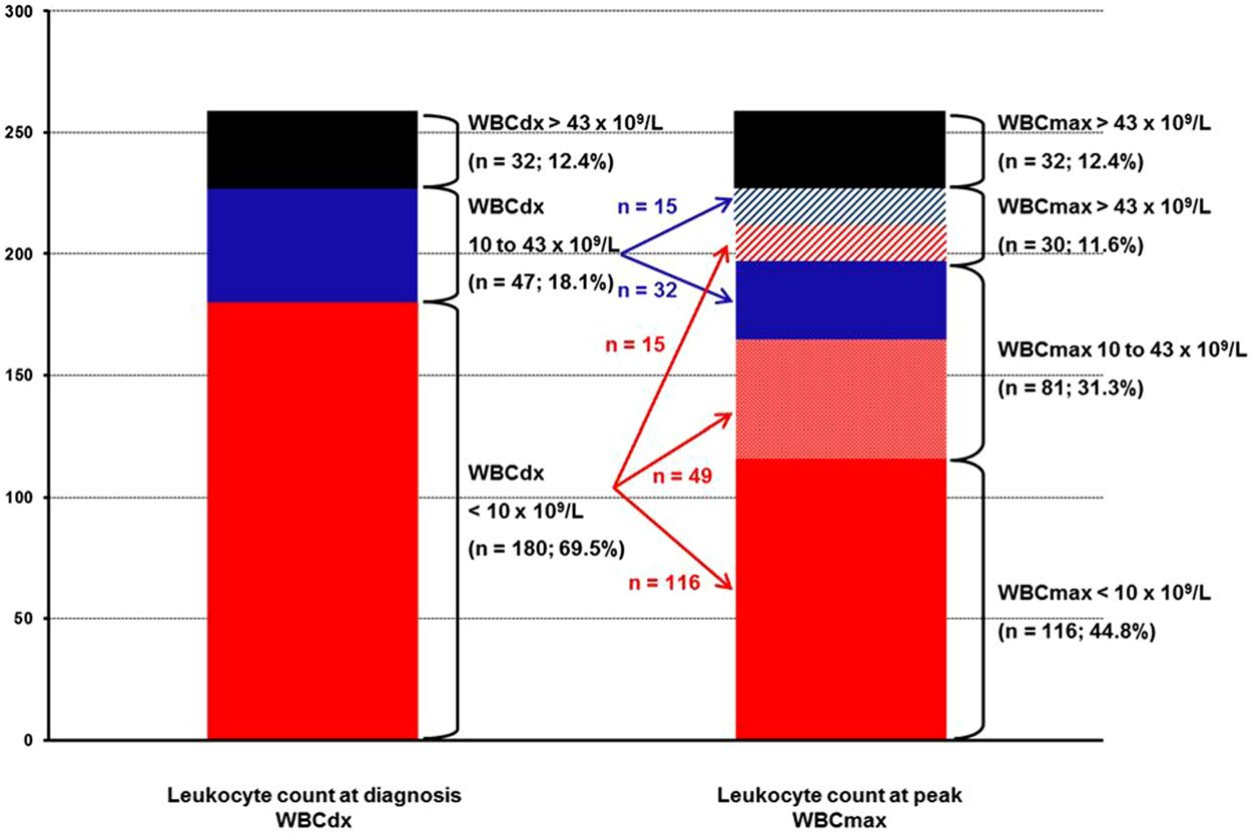
Proportion of patients whose WBC count changed from diagnosis (WBCdx) to peak (WBCmax) during the initial treatment period (right bars). Black bar (n = 32), sustained hyperleukocytosis (both WBCdx and WBCmax >43 × 10^9^/L); blue diagonal lines (n = 15), progressive leukocytosis (WBCdx 10 to 43 × 10^9^/L to WBCmax >43 × 10^9^/L); red diagonal lines (n = 15), progressive leukocytosis (WBCdx <10 × 10^9^/L to WBCmax >43 × 10^9^/L); blue bar (n = 32), sustained leukocytosis (both WBCdx and WBCmax 10 to 43 × 10^9^/L); pink bar (n = 49), progressive leukocytosis (WBCdx <10 × 10^9^/L to WBCmax 10 to 43 × 10^9^/L); red bar, sustained low WBC (both WBCdx and WBCmax <10 × 10^9^/L).

According to the Sanz-risk scoring, the leukocyte count cut-off for high-risk patients was WBCdx >10 × 10^9^/L. In a receiver operating characteristic (ROC) curve analysis, we found a significant leukocyte count cut-off for predicting both early death and DS at the level of >10 × 10^9^/L for WBCdx and >43 × 10^9^/L for WBCmax, respectively. Using those levels, we grouped patients by their leukocyte count increases from their diagnostic levels (Fig. 2). Group analysis using diagnostic leukocyte counts and the increment level was performed except for patients with WBCdx >43 × 10^9^/L, who already showed high leukocyte counts. The first leukocyte increment group contained patients with WBCdx <10 × 10^9^/L (n = 180), and the second group contained patients with WBCdx 10 to 43 × 10^9^/L (n = 47). Then, we identified 3 increment subgroups in the WBCdx <10 × 10^9^/L group — subgroup 1, sustained low, WBCmax <10 × 10^9^/L (red, n = 116); subgroup 2, increment to WBCmax 10 to 43 × 10^9^/L (pink, n = 49); subgroup 3, increment to WBCmax >43 × 10^9^/L (red diagonal, n = 15). Next, we identified 2 subgroups from the WBCdx 10 to 43 × 10^9^/L group — subgroup 1, sustained WBCmax 10 to 43 × 10^9^/L (blue, n = 32); subgroup 2, increment to WBCmax >43 × 10^9^/L (blue diagonal, n = 15).

### Progressive hyperleukocytosis affecting early death and DS

**Figure 3:**
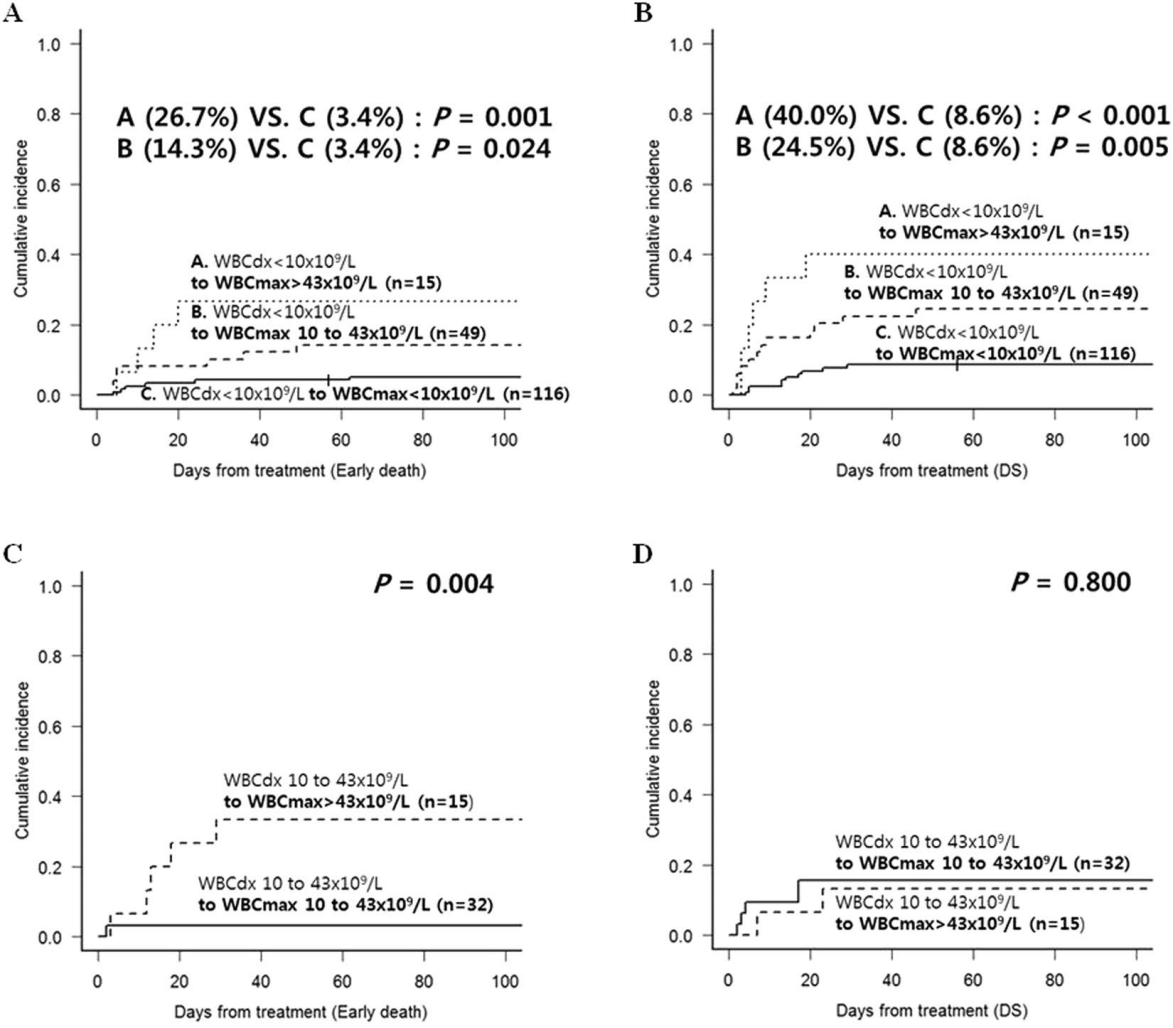
The different early outcomes according to leukocytosis progress. Subgroup analyses were performed in the group with WBCdx <10 × 10^9^/L (red bar [Lt.] and 3 red arrows in Fig. 2) and the group with WBCdx 10 to 43 × 10^9^/L (blue bar [Lt.] and 2 blue arrows in Fig. 2). (**A**) Early death rates in the 3 subgroups with progression from WBCdx <10 × 10^9^/L. (**B**) DS rates in the 3 subgroups with progression from WBCdx <10 × 10^9^/L. (**C**) Early death rates in the 2 subgroups with progression from WBCdx 10 to 43 × 10^9^/L. (**D**) DS rates in the 2 subgroups with progression from WBCdx 10 to 43 × 10^9^/L.

We calculated the cumulative incidence of early death and DS in the 2 large groups and their subgroups. In the low Sanz-risk group — WBCdx <10 × 10^9^/L (Fig. 3A,B), the increment to WBCmax 10 to 43 × 10^9^/L and increment to WBCmax >43 × 10^9^/L subgroups had increased 8-week early death rates (14.3% and 26.7%, *p* = 0.024 and *p* = 0.001, respectively) and DS rates (24.5% and 40.0%, *p* = 0.005 and *p* < 0.001, respectively) compared with patients with sustained WBCmax <10 × 10^9^/L (3.4% for early death and 8.6% for DS). In the WBCdx 10 to 43 × 10^9^/L group (Fig. 3C,D), the increment to WBCmax >43 × 10^9^/L subgroup had a higher 8-week early death rate than patients with sustained WBCmax 10 to 43 × 10^9^/L (33.3% vs. 3.1%, *p* = 0.004), but the DS rate did not differ significantly between the two subgroups (13.3% vs. 15.6%, *p* = 0.800).

### Maximum leukocyte count (WBCmax) and treatment outcomes

**Figure 4:**
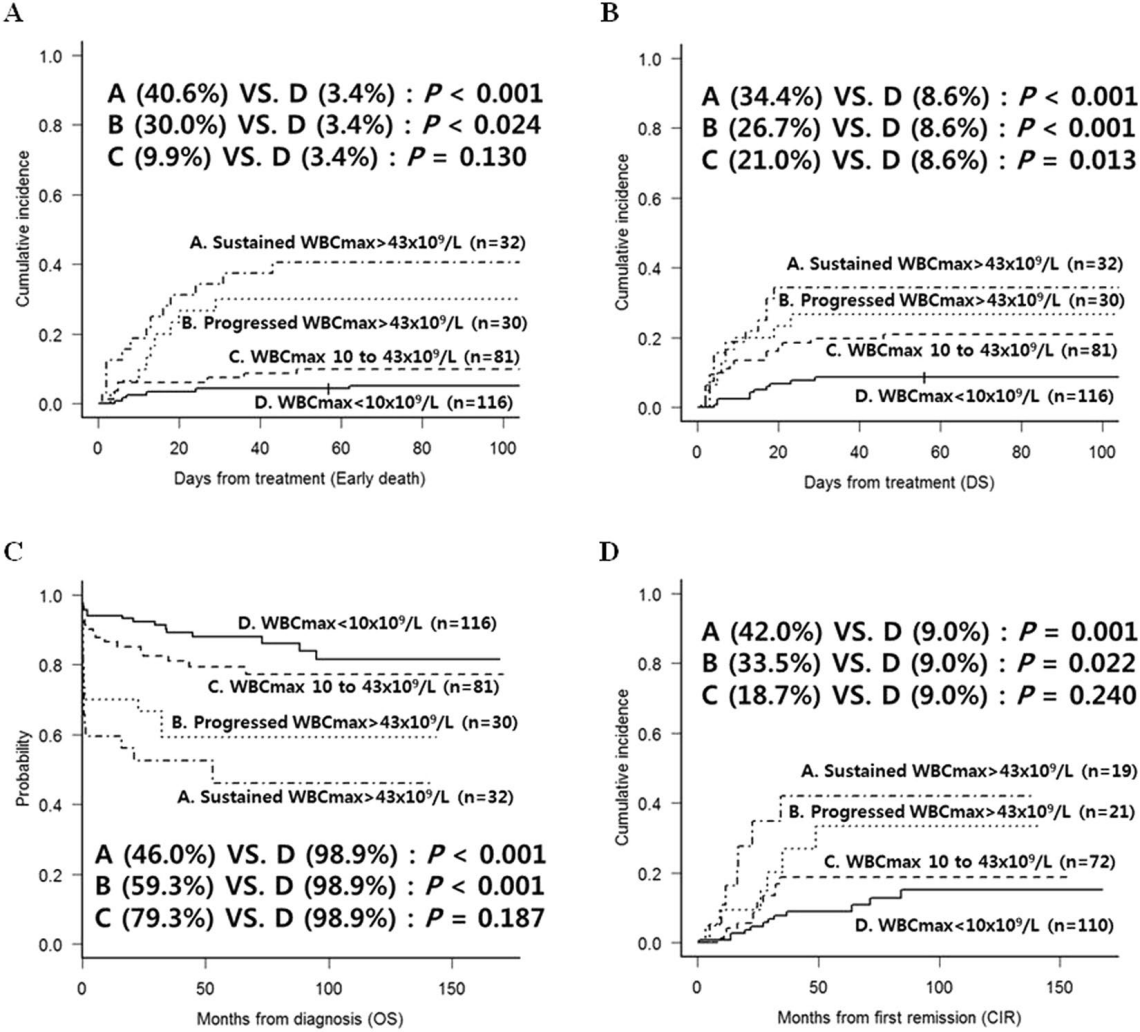
Survival outcomes of 4 subgroups stratified by the maximum leukocyte count (WBCmax) and progression of hyperleukocytosis. (**A**) Early death rates. (**B**) DS rates. (**C**) OS. (**D**) CIR.

Next, we stratified patients into 4 subgroups according to their maximum leukocyte count (WBCmax). Right bar in the previous Fig. 2 shows the proportion of patients in each subgroup — WBCmax <10 × 10^9^/L (n = 116), WBCmax 10 to 43 × 10^9^/L (n = 81), progressed WBCmax >43 × 10^9^/L (n = 30), and sustained WBCmax >43 × 10^9^/L (n = 32). The 8-week early death rates for the 4 subgroups were 3.4%, 9.9%, 30.0%, and 40.6%, respectively (Fig. 4A), and the DS rates were 8.6%, 21.0%, 26.7%, and 34.4%, respectively (Fig. 4B). We also calculated the long-term survival outcomes with consideration of the early outcomes. The estimated 5-year OS rates were 98.9% for the WBCmax <10 × 10^9^/L group, 79.3% for the WBCmax 10 to 43 × 10^9^/L group, 59.3% for the progressed WBCmax >43 × 10^9^/L group, and 46.0% for the sustained WBCmax >43 × 10^9^/L group (Fig. 4C), and the 5-year CIR rates were 9.0% for the WBCmax <10 × 10^9^/L group, 18.7% for the WBCmax 10 to 43 × 10^9^/L group, 33.5% for the progressed WBCmax >43 × 10^9^/L group, and 42.0% for the sustained WBCmax >43 × 10^9^/L group (Fig. 4D). The multivariate analysis showed that early death correlated with age older than 40 years (HR = 2.38, 95% CI: 1.1–5.1, *p* = 0.026), progressed WBCmax >43 × 10^9^/L (HR = 3.72, 95% CI: 1.4–9.8, *p* = 0.008), and sustained WBCmax >43 × 10^9^/L (HR = 5.38, 95% CI: 2.2–13.2, *p* < 0.001). A higher DS rate correlated with an antithrombin III (ATIII) level ≤ 100% (HR = 2.51, 95% CI: 1.1–5.9, *p* = 0.036), WBCmax 10 to 43 × 10^9^/L (HR = 3.06, 95% CI: 1.4–6.6, *p* = 0.004), progressed WBCmax >43 × 10^9^/L (HR = 3.29, 95% CI: 1.3–8.2, *p* = 0.011), and sustained WBCmax >43 × 10^9^/L (HR = 5.79, 95%CI: 2.5–13.4, *p* < 0.001) compared with patients with WBCmax <10 × 10^9^/L (Table 2).

**Table 2:** Multivariate analysis of affecting factors for early death and differentiation syndrome.

Variables	Early death				Differentiation syndrome (DS)			
	Univariate		Multivariate		Univariate		Multivariate	
	8-week death	*p*	HR (95% CI)	*p*	8-week DS	*p*	HR (95% CI)	*p*
**Age at diagnosis**								
<40 years (n = 111)	8.1%	0.033*			18.2%	0.933		
≥40 years (n = 148)	17.6%		2.38 (1.1–5.1)	0.026*	17.6%			
**Platelet counts at diagnosis**								
<30 × 10^9^/L (n = 124)	17.7%	0.061			21.8%	0.110		
≥30 × 10^9^/L (n = 135)	9.6%				14.2%			
***FLT3*** **mutation**								
No *FLT3* mutation (n = 118)	6.8%	0.006*			16.9%	0.189		
*FLT3*-ITD or –TKD (n = 46)	21.7%				26.1%			
**BCR subtype**								
BCR1 (n = 120)	8.3%	0.018*			17.6%	0.243		
BCR3 (n = 70)	20.0%				24.3%			
**Leukocyte count at diagnosis**								
<10.0 × 10^9^/L (n = 180)	8.3%	<0.001*			15.6%	0.026*		
10.0 − 43.0 × 10^9^/L (n = 47)	12.8%				14.9%			
>43.0 × 10^9^/L (n = 32)	40.6%				34.4%			
**Leukocyte count at peak (WBCmax)**								
<10.0 × 10^9^/L (n = 116)	3.4%	<0.001*	1.0		8.6%	0.001*	1.0	
10.0 − 43.0 × 10^9^/L (n = 81)	9.9%		1.12 (0.3–3.4)	0.843	21.0%		3.06 (1.4–6.6)	0.004*
Progressed > 43.0 × 10^9^/L (n = 30)	30.0%		3.72 (1.4–9.8)	0.008*	26.7%		3.29 (1.3–8.2)	0.011*
Sustained > 43.0 × 10^9^/L (n = 32)	40.6%		5.38 (2.2–13.2)	<0.001*	34.4%		5.79 (2.5–13.4)	<0.001*
**D-dimer at diagnosis**								
≤20 mg/L (n = 132)	3.0%	<0.001*			15.2%	0.238		
>20 mg/L (n = 127)	24.4%		6.39 (2.2–18.4)	0.001*	20.6%			
**ATIII level at diagnosis**								
≤100% (n = 191)	16.8%	0.011*			21.1%	0.023*	2.51 (1.1–5.9)	0.036*
>100% (n = 68)	4.4%				8.8%			
**Fibrinogen level at diagnosis**								
<140 mg/dL (n = 139)	19.4%	0.002*			20.1%	0.300		
≥140 mg/dL (n = 120)	6.7%				15.1%			

Abbreviation: CIR, cumulative incidence of relapse; HR, hazard ratio; *FLT3*, Fms-like tyrosine kinase 3; ITD, internal tandem duplication; TKD, tyrosine kinase domain; BCR, breakpoint cluster region; ATRA, all-trans retinoic acid; ATIII, Antithrombin III.

## Discussion

In this study, we have found good survival outcomes from our analysis of 259 APL patients treated with ATRA and anthracycline-based chemotherapy based on the AIDA protocol^1,18,19^. As we experienced, except for the 35 (13.5%) patients who died within 8 weeks, NRM was low, and most long-term failures were caused by APL relapses: 5-year NRM was 1.2% and 5-year CIR was 17.1%. Most early deaths were caused by significant bleeding complications, including intracranial hemorrhage and alveolar hemorrhage, followed by multiorgan failure.

We also identified the cumulative incidence of DS, which occurred in 46 patients (17.8%). Of those, severe DS was observed in 9 (19.5%), and 4 of them died of progressive organ failure caused by the severe DS. This value is lower than in a previous large cohort study^13^; most of our DS cases were safely manageable with intravenous dexamethasone and immediate cessation of ATRA. However, we might have missed some patients with severe DS that was misdiagnosed as a bleeding complication, such as alveolar hemorrhage. Of the 35 early deaths in our cohort, 16 patients suffered from alveolar hemorrhage.

Sanz-risk scoring suggests that the leukocyte count prior to therapy is the prognostic factor most relevant to relapse and survival outcomes^17,20^. Although little was previously reported about factors predictive of early death and DS, a study by Vahdat *et al*. showed that peak leukocyte count correlated with DS^16^, and some studies have suggested that an abnormal creatinine level, male sex, old age, and hyperleukocytosis at diagnosis are related to early death^14,17^. However, no factors have been consistently shown to affect early complications.

We stratified patients into groups by their diagnostic leukocyte count (WBCdx), leukocyte increment ratio, and maximum leukocyte count (WBCmax). Classic Sanz-risk scoring suggests that a WBCdx higher than 10 × 10^9^/L is a significant cut-off for long-term outcomes. Our ROC analysis in this study found that both a WBCdx and WBCmax higher than 43 × 10^9^/L correlated significantly with early death and DS. Overall, we first identified changing outcomes according to 3 levels of leukocyte increment beginning with a WBCdx lower than 10 × 10^9^/L. Progression to both WBCmax 10 to 43 × 10^9^/L and WBCmax >43 × 10^9^/L correlated with a significantly higher incidence of DS and early death compared with patients with a WBCmax <10 × 10^9^/L. In addition, the 2 subgroups with progressive hyperleukocytosis showed significantly higher CIR rates and inferior OS even though they had low leukocyte counts at diagnosis. This might be the first evidence that progressive leukocytosis (as reflected by WBCmax) is a more relevant factor in predicting early and long-term survival outcomes than the leukocyte count prior to therapy. At this point, we question whether ATRA or ATO should be started in patients with hyperleukocytosis and wonder whether cytoreduction or leukapheresis is more important for patients with hyperleukocytosis to reduce the rates of DS and early death. However, no definite guidelines discuss those issues because high-risk APL patients are rare, and individual differences make it difficult to draw definite conclusions for standard management strategies. Nonetheless, the role of prophylactic dexamethasone should be studied in the near future.

The multivariate analysis also revealed that old age correlated with early death, and a low ATIII level correlated with DS. For ATIII, our further analysis revealed that ATIII >100% correlated with significantly lower incidence of DS, even in patients with WBCmax >43 × 10^9^/L. Because we can support ATIII in patients using disseminated intravascular coagulation and a supportive management strategy, ATIII and transfusions could be adjusted in future prospective trials. In old age, especially for people with comorbidity, intensive chemotherapy might be toxic, causing bleeding complications and sepsis. Therefore, using ATO plus ATRA could be an important alternative for elderly APL patients^8,10,11,15^. A randomized phase III trial demonstrated that ATRA plus ATO is at least non-inferior and could be superior to ATRA plus chemotherapy in low- to intermediate-risk APL^8^. In high-risk APL, a more tailored therapy using ATO and attenuating conventional chemotherapy is needed to reduce the early death rate in elderly patients^21^.

We checked for the *FLT3*-ITD mutation and break point isoforms (*BCR1* and *BCR3*) in the current study, and we found that the *FLT3* mutation and *BCR3* isoform correlated with high leukocyte counts and early death. However, that analysis was many missing data points; in the multivariate analysis, no molecular parameter correlated with early death or DS. Nonetheless, other recent studies have revealed that cell surface markers in APL blasts, such as CD34, CD56, T-cell antigen CD2, the *BCR3* isoform, and the *FLT3*-ITD mutation are associated with hyperleukocytosis and a high risk of relapse^22^. In addition, the presence of the *FLT3*-ITD mutation correlates with an increased occurrence of thrombotic events, increased leukocyte count, immature cell phenotypes such as CD34 and CD2, and the *BCR3* isoform. Our data also showed a relationship between the *FLT3* mutation and *BCR3* isoform, but in our data, WBCmax showing progressive leukocytosis was the most important independent factor for predicting early complications and long-term survival outcomes.

Although our current results are from a retrospective study, all enrolled patients were treated with the same management strategy for a long period, which could minimize possible biases. Unfortunately, because the standard treatment for APL has changed to a combination therapy using ATO, these results might no longer be applicable, so our results need further validation. In conclusion, our data show that a WBCmax indicating progressive leukocytosis correlated better with early death and DS than the Sanz-risk criteria. The role of dexamethasone prophylaxis, antithrombin III, and a cytoreduction strategy should be evaluated in specific patient subsets to reduce early events in APL. In addition, being in the high-increment WBCmax group also correlated with a high risk of relapse, so early intervention with close monitoring and preventive management might improve survival outcomes^23^.

## Methods

### Study population

We initially identified 259 adult APL patients with a median age of 42 years (range; 15–72 years) from 2002 to 2014. We confirmed APL with a chromosomal analysis and additional *PML-RARA* reverse transcriptase polymerase chain reaction (RT-PCR), followed by reverse transcription quantitative PCR (RT-qPCR). To detect the karyotypes, at least 20 metaphases from BM cells were analyzed by the GTG banding method after 24 or 48 h of unsynchronized culture, and the International System for Cytogenetic Nomenclature was used as a guideline for classification^24^. We also identified the presence of additional chromosomal abnormalities. The purpose and experimental protocols of this research were approved by the Institutional Review Board and Ethics Committee guidelines of the Catholic Medical Center (KC15RISI0862) and the principles of the Declaration of Helsinki. Informed consent of all patients was obtained according to established guidelines. For patients younger than 18 years, consent was obtained from their legal guardians.

### Treatment strategy and supportive care

Except for 1 woman, all patients received ATRA (45 mg/m^2^/day) divided into 2 daily doses immediately upon suspicion of APL or with morphological evidence from a BM aspiration study. We performed leukapheresis whenever leukocyte counts exceeded 50 × 10^9^/L, and some patients with hyperleukocytosis were treated with hydroxyurea, cytarabine, and prophylactic dexamethasone. Available blood products were vigorously administered with frequent monitoring. The target level for transfusion was higher than 50 × 10^9^/L for platelets, higher than 120 mg/dL for fibrinogen, higher than 70% for prothrombin time, and higher than 70% for ATIII. Most patients were treated with induction chemotherapy using idarubicin (12 mg/m^2^, days 1, 3, 5, 7) based on the AIDA protocol, as previously reported^1,18,19^, but some patients (n = 13) with serious infectious complications or comorbidity were treated with a compassionate program of ATO, and some (n = 26) were treated with ATRA alone. After achievement of hematological CR, all patients received the same consolidation chemotherapy consisting of three courses in combination with 15 days of ATRA as follows — course 1, idarubicin (7 mg/m^2^, days 1–4); course 2, mitoxantrone (10 mg/m^2^, days 1–4); and course 3, idarubicin (12 mg/m^2^, day 1–2) — which was based on the LPA99 protocol from PETHEMA^6,25^. After completion of consolidation, patients received the following maintenance therapy for 2 years: 6-mercaptopurine (50 mg/m^2^/day) and ATRA for 15 days every 3 months. Our maintenance was a modified regimen (by exclusion of methotrexate) based on a protocol previously reported^6,19,25^. When DS occurred, we promptly discontinued ATRA and started dexamethasone (10 mg twice daily), with strict control of volume overloading using diuretics or renal replacement therapy.

### Molecular studies

The molecular studies were performed from the BM samples collected at diagnosis, 1 month after induction and consolidation chemotherapies, and every 3 months after maintenance treatment. *PML-RARA*, the representative marker for measurable residual disease, was detected by a multiplex RT-PCR screening assay using a HemaVision kit (DNA Technology, Aarthus, Denmark), and quantification was performed using the RT-qPCR method (Real-Q *PML-RARA* quantification kit, Biosewoom, Korea) with a sensitivity of 5.0 log (10^−5^). The RQ-PCR level represented the ratios of *PML-RARA* normalized to the expression of the reference gene, *ABL1* (1.0 × 10^4^). The *FLT3* mutation was detected using multiplex allele-specific RT-PCR (ABSOULTE *FLT3 TKD/ITD* RT-PCR, Biosewoom, Korea). Because those molecular studies became available in 2006, we could not obtain molecular data for patients treated before that.

### Primary endpoint and definitions

The primary endpoint of the current study was to determine predictive factors that affect early complications, early death, and DS, in APL patients. Therefore, we tried to identify the prognostic value of progressive hyperleukocytosis after ATRA administration during initial treatment. We checked the diagnostic level (WBCdx) and maximum level (WBCmax) of the leukocyte counts during the initial treatment period and identified subgroups with progressive hyperleukocytosis, i.e., those with a low WBCdx <10 × 10^9^/L whose WBCmax was significantly higher than that. The definition of early death was all patients who died within 8 weeks of treatment. DS was diagnosed as the presence of at least 2 of the following — dyspnea, unexplained fever, sudden weight gain greater than 5 kg, unexplained hypotension, acute renal failure, or a chest radiograph demonstrating pulmonary infiltration or pleuropericardial effusion^4,13^. Any patient showing 4 or more of those features was considered to have severe DS, and those with fewer than 4 were classified as having moderate DS.

### Statistical analysis

All categorical variables were compared by Fisher’s exact tests, and continuous variables were assessed with the Mann-Whitney *U* test. To determine a significant cut-off level at which WBCmax could predict early death or DS, we used a ROC curve analysis. OS and DFS rates were calculated using Kaplan-Meier survival analyses, and the log-rank test was used to evaluate differences between subgroups. CIR and NRM rates were calculated using cumulative incidence estimations that treated non-relapse deaths and relapse as competing risks for CIR and NRM, respectively, and they were compared using the Gray test^26^. Early death and DS rates were also calculated by cumulative incidence estimations that used untreated patients and early deaths as competing risks, respectively. Multivariate analyses for the cumulative incidence of early death and DS were calculated using a Fine-Gray proportional hazard regression model. All statistical analyses were performed using ‘R’ software (ver. 2.15.1, R Foundation for Statistical Computing, 2012). Statistical significance was set at a *p* value < 0.05.
